# Comparative molecular cytogenetic characterization of five wild *Vigna* species (Fabaceae)

**DOI:** 10.3897/CompCytogen.v14i2.51154

**Published:** 2020-06-26

**Authors:** Chao-Wen She, Ying Mao, Xiang-Hui Jiang, Chun-Ping He

**Affiliations:** 1 Key Laboratory of Research and Utilization of Ethnomedicinal Plant Resources of Hunan Province, Huaihua University, Huaihua, Hunan, 418008, China Huaihua University Huaihua China; 2 Key Laboratory of Xiangxi Medicinal Plant and Ethnobotany of Hunan Higher Education, Huaihua University, Huaihua, Hunan, 418008, China Huaihua University Huaihua China; 3 College of Biological and Food Engineering, Huaihua University, Huaihua, Hunan, 418008, China Huaihua University Huaihua China; 4 College of Chemistry and Material Engineering, Huaihua University, Huaihua, Hunan, 418008, China Huaihua University Huaihua China

**Keywords:** *Vigna* species, karyotype, fluorochrome banding, fluorescence *in situ* hybridization (FISH), ribosomal RNA gene (rDNA)

## Abstract

To extend our knowledge on karyotype variation of the genus *Vigna* Savi, 1824, the chromosomal organization of rRNA genes and fluorochrome banding patterns of five wild *Vigna* species were studied. Sequential combined PI (propidium iodide) and DAPI (4',6-diamidino-2-phenylindole) (CPD) staining and fluorescence *in situ* hybridization (FISH) with 5S and 45S rDNA probes were used to analyze the karyotypes of *V. luteola* (Jacquin, 1771) Bentham, 1959, *V. vexillata* (Linnaeus, 1753) A. Richard, 1845, *V. minima* (Roxburgh, 1832) Ohwi & H. Ohashi, 1969, *V. trilobata* (Linnaeus, 1753) Verdcourt, 1968, and *V. caracalla* (Linnaeus, 1753) Verdcourt,1970. For further phylogenetic analysis, genomic *in situ* hybridization (GISH) with the genomic DNA of *V. umbellata* (Thunberg, 1794) Ohwi & H.Ohashi, 1969 onto the chromosomes of five wild *Vigna* species was also performed. Detailed karyotypes were established for the first time using chromosome measurements, fluorochrome bands, and rDNA-FISH signals. All species had chromosome number 2n = 2x = 22, and symmetrical karyotypes that composed of only metacentric or metacentric and submetacentric chromosomes. CPD staining revealed all 45S rDNA sites in the five species analyzed, (peri)centromeric GC-rich heterochromatin in *V. luteola*, *V. trilobata* and *V. caracalla*, interstitial GC-rich and pericentromeric AT-rich heterochromatin in *V. caracalla*. rDNA-FISH revealed two 5S loci in *V. caracalla* and one 5S locus in the other four species; one 45S locus in *V. luteola* and *V. caracalla*, two 45S loci in *V. vexillata* and *V. trilobata*, and five 45S loci in *V. minima*. The karyotypes of the studied species could be clearly distinguished by the karyotypic parameters, and the patterns of the fluorochrome bands and the rDNA sites, which revealed high interspecific variation among the five species. The *V. umbellata* genomic DNA probe produced weak signals in all proximal regions of *V. luteola* and all (peri)centromeric regions of *V. trilobata*. The combined data demonstrate that distinct genome differentiation has occurred among the five species during evolution. The phylogenetic relationships between the five wild species and related cultivated species of *Vigna* are discussed based on our present and previous molecular cytogenetic data.

## Introduction

The genus *Vigna* Savi, 1824, belonging to the tribe Phaseoleae of the family Fabaceae, includes over 100 species distributed throughout the Old and New Worlds (Schrire 2005). Taxonomically, this genus was divided into seven subgenera by Maréchal et al. (1981), among which subg. Vigna Savi, 1876 and subg. Ceratotropis (Piper) Verdcourt, 1969 includes the seven economically important crop species, *V. unguiculata* (Linnaeus, 1753) Walp, 1842, *V. subterranea* (Linnaeus, 1753) Verdcourt, 1980, *V. aconitifolia* (Jacquin, 1771) Maréchal, 1969, *V. angularis* (Willdenow, 1800) Ohwi & H. Ohashi, 1969, *V. mungo* (Linnaeus, 1753) Hepper, 1956, *V. radiata* (Linnaeus, 1753) R. Wilczek, 1954, and *V. umbellata* (Thunberg, 1794) Ohwi & H.Ohashi, 1969 (Smartt 1990). An understanding of the phylogenetic relationships among the cultigens and their wild relatives is helpful for developing crop improvement tools and gene transfer strategies. A lot of DNA-level studies, such as analyses of the internal transcribed spacers (ITS) of rDNA (Doi et al. 2002; Goel et al. 2002; Saini et al. 2008; Delgado-Salinas et al. 2011; She et al. 2015; Raveenadar et al. 2018), the intergenic spacer (IGS) of 5S rDNA (Saini and Jawali 2009), plastid DNA sequences (Doi et al. 2002; Tun and Yamaguchi 2007; Javadi et al. 2011; Delgado-Salinas et al. 2011; Raveenadar et al. 2018), and DNA amplification fingerprinting (Simon et al. 2007), have been conducted to reveal the phylogenetic relationships among *Vigna* species. A molecular cytogenetic investigation has also been performed to help clarify the phylogenetic relationships among the seven cultivated *Vigna* species (She et al. 2015). However, comparative molecular cytogenetic study on the phylogenetic relationships between the cultivated *Vigna* species and closely related wild species has not been conducted till now.

The chromosomes of *Vigna* species were rather small in size and poorly morphologically differentiated (Guerra et al. 1996), resulting in the difficulty of distinguishing chromosomes. To date, only about twenty wild *Vigna* species were cytogenetically studied, and these studies were mostly restricted to chromosome counts and karyomorphological descriptions (Sen and Bhowal 1960; Joseph and Bouwkamp 1978; Rao and Chandel 1991; Galasso et al. 1993, 1996; Venora and Saccardo 1993; Venora et al. 1999; Shamurailatpam et al. 2012, 2015, 2016), which could not provide reliable information on genome evolution among related species. Although many molecular cytogenetic studies have been conducted for the cultivated *Vigna* species using fluorescence *in situ* hybridization (FISH) with 5S and 45S ribosomal genes (rDNAs; Galasso et al. 1995, 1998; Guerra et al. 1996; Khattak et al. 2007; de A Bortoleti et al. 2012; Choi et al. 2013; She et al. 2015), but only one wild *Vigna* species has been molecular-cytogenetically investigated so far (Choi et al. 2013).

FISH mapping of repetitive DNA sequences such as 5S and 45S rDNAs can not only generate useful landmarks for chromosome identification but can also provide valuable information on the evolutionary relationships between related species (e.g. Moscone et al. 1999; Zhang and Sang 1999; Hasterok et al. 2001; de Moraes et al. 2007; Hamon et al. 2009; Robledo et al. 2009; Wolny and Hasterok 2009; She et al. 2015; Li et al. 2016; Amosova et al. 2017; Maragheh et al. 2019). To date, the number and position of rDNA loci have been determined in more than 1600 plant species with FISH (Garcia et al. 2014). These studies showed that the number and position of the 5S and 45S rDNAs were usually characteristics of a given species or genus (e.g. Moscone et al. 1999; Hasterok et al. 2001; Chung et al. 2008; Hamon et al. 2009; Robledo et al. 2009; Wolny and Hasterok 2009; She et al. 2015; Li et al. 2016; Maragheh et al. 2019). Fluorochrome banding techniques using double fluorescent dyes such as CMA3 (chromomycin A3) /DAPI (4’,6-diamidino-2-phenylindole) staining, and PI (propidium iodide)/ DAPI staining (called CPD staining) was used to localize the chromosome regions that are rich in GC and AT base pairs simultaneously, providing effective identifying markers for chromosomes, and revealing characteristic heterochromatin distribution along chromosomes (She et al. 2006; de Moraes et al. 2007; de A Bortoleti et al. 2012; She and Jiang 2015; She et al. 2015, 2017; Tang et al. 2019).

Detailed karyotypes can be constructed using the dataset of rDNA-FISH signals, fluorochrome bands and chromosome measurements, which reveals the genome organization of a plant species at chromosome level and is valuable in investigating the evolutionary relationships between related species (e.g. Moscone et al. 1999; de Moraes et al. 2007; Hamon et al. 2009; Robledo et al. 2009; Mondin and Aguiar-Perecin 2011; She and Jiang 2015; She et al. 2015, 2017; Zhang et al. 2015; Amosova et al. 2017; Tang et al. 2019) and helpful to integrate the genetic and physical maps of a plant species (Fuchs et al. 1998; Fonsêca et al. 2010). Comparative genomic *in situ* hybridization (cGISH) is a modification of the GISH technology in which the labelled total genomic DNA of one species is hybridized to the chromosomes of another species without the competitive DNA. It generates hybridization signals in the chromosomal regions of conserved repetitive DNA sequences. Therefore, it can directly identify the genome relationships among related species (Falistocco et al. 2002; Wolny and Hasterok 2009; She et al. 2015, 2017; Amosova et al. 2017).

In the present study, molecular cytogenetic characterization of five wild *Vigna* species, *V. luteola*, *V. vexillata*, *V. minima*, *V. trilobata* and *V. caracalla* was conducted using sequential CPD staining and dual color FISH with 5S and 45S rDNA probes. Detailed karyotypes of the five species were established using a combination of chromosome measurements, fluorochrome bands, and rDNA-FISH signals. Six different parameters of karyotype asymmetry were calculated for the elucidation of karyotype variation among these species. cGISH with *V. umbellata* genomic DNA probe onto the somatic chromosomes of the five species, the method that was applied in the molecular-cytogenetic study on the seven cultivated *Vigna* species (She et al. 2015), was also performed. The datasets were assessed to gain insights into the genome differentiation and phylogenetic relationships among the five wild and seven cultivated *Vigna* species.

## Material and methods

### Plant materials and DNA extraction

Seeds of *V. luteola* (Jacquin, 1771) Bentham, 1959 (PI 406329), *V. vexillata* (Linnaeus, 1753) A.Richard, 1845 (PI 406428, Origin traced to PI 225934), *V. minima* (Roxburgh, 1832) Ohwi & H. Ohashi, 1969 (PI 483081), *V. trilobata* (Linnaeus, 1753) Verdcourt, 1968 (PI 286306), *V. caracalla* (Linnaeus, 1753) Verdcourt, 1970 (Synonym of *Cochliasanthus caracalla* (Linnaeus, 1753) Trew, 1764; PI 146800), and *V. umbellata* (Thunberg, 1794) Ohwi & H. Ohashi, 1969 (PI 208460) were obtained from the U.S. National Plant Germplasm System. Genomic DNA of *V. umbellata* was isolated from young leaves using Rapid Plant Genomic DNA Isolation Kit (Sangon Biotech, Shanghai, China).

### Chromosome preparation

Mitotic metaphase chromosome spreads were prepared as previously described with minor modification (She et al. 2006). In brief, seeds were germinated on moistened filter paper in the dark at 28 °C. Root tips were harvested and treated in saturated α-bromonaphthalene at 28 °C for 2.0 h, and then fixed in methanol-glacial acetic acid (3:1) at 4 °C. The fixed root tips were thoroughly rinsed in double-distilled water and digested in an enzymatic solution composed of 1% cellulase RS (Yakult Pharmaceutical Industry Co., Ltd. Tokyo, Japan), 1% pectolyase Y23 (Yakult Pharmaceutical Industry Co., Ltd. Tokyo, Japan) in citric buffer (0.01 mM citric acid-sodium citrate, pH 4.5) at 28 °C for 100–120 mins. The digested root tips were gently placed on a glass slide with methanol-glacial acetic acid (3:1) and dissected thoroughly by using fine-pointed forceps. Then, the slides were flame-dried. The slides with well-spread somatic metaphase chromosomes were screened under phase contrast microscope and stored at -20 °C until used.

### CPD staining

CPD staining followed the procedure described by She et al. (2006). Briefly, chromosome preparations were treated with RNase A and pepsin and then stained with a mixture of 0.6 µg·ml^-1^PI and 3 µg·ml^-1^DAPI in a 30% (v/v) solution of Vectashield H100 (Vector Laboratories, Burlingame, US) for at least 30 min in the dark at room temperature. Slides were examined under an Olympus BX60 epifluorescence microscope. Separate images from UV and green filters were captured using a cooled CCD camera (CoolSNAP EZ; Photometrics, Tucson, US) controlled using METAMORPH software (Molecular Devices, California, US). DAPI and PI grey scale images of the same plate were merged to produce a CPD image. Final images were optimized for contrast and brightness using ADOBE PHOTOSHOP version 8.01.

### Probe DNA labelling

A 45S rDNA clone containing a 9.04-kb tomato 45S rDNA insert (Perry and Palukaitis 1990) and a pTa794 clone containing a 410-bp BamHI fragment of wheat 5S rDNA (Gerlach and Bedbrook 1979) were used as probes to localize the two ribosomal RNA genes. The 45S clone was labeled with biotin-16-dUTP, and the 5S clone and the *V. umbellata* genomic DNA were labeled with digoxigenin-11-dUTP, using Nick Translation Kit (Roche Diagnostics, Mannheim, Germany).

### Fluorescence *in situ* hybridization

FISH with the 5S and 45S rDNA probes, and cGISH with *V. umbellata* genomic DNA probe were performed after CPD staining on the same slides. The slides previously stained by CPD were washed in 2× SSC, twice for 15 min each, dehydrated through an ethanol series (70%, 90%, and 100%, 5 min each) and then used for hybridization. The *in situ* hybridization methodology followed the protocol described by She et al. (2015). The biotin-labelled probe was detected using Fluorescein Avidin D (Vector Laboratories, Burlingame, USA). The digoxigenin-labeled probe was detected by anti-digoxigenin-rhodamine (Roche Diagnostics, Mannheim, Germany). The preparations were counterstained and mounted with 3 µg ml^−1^DAPI in 30% (v/v) Vectashield H-1000 and examined under the epifluorescence microscope mentioned above. Grey-scale images were digitally captured using METAMORPH software with UV, blue and green filters for DAPI, fluorescein, and rhodamine, respectively. The images were then merged and edited with ADOBE PHOTOSHOP version 8.01.

### Karyotype analysis

The karyotyping methodology followed that described by She et al. (2015). Five metaphase plates of each species were measured using ADOBE PHOTOSHOP version 8.01. The chromosome relative lengths (RL, % of haploid complement), arm ratios (AR = long arm/short arm), size of the fluorochrome band, and percent distance from the centromere to the rDNA site were calculated. The total length of the haploid complement (TCL; i.e. the karyotype length) was measured using five metaphase cells with the highest condensation degree. The arm ratio was used to classify the chromosomes according to the system described by Levan et al. (1964). Idiograms were drawn based on measurements, fluorochrome bands, and rDNA-FISH signals. The chromosomes were organized in decreasing order. Karyotype asymmetry was determined using the mean centromeric index (CI), the intrachromosomal asymmetry index (A1), the interchromosomal asymmetry index (A2) (Romero Zarco 1986), the ratio of long arm length in chromosome set to total chromosome length in set (As K%) (Arano 1963), the asymmetry index (AI) (Paszko 2006), and the categories of Stebbins (1971).

## Results

### General karyotype features

**Figure 1.**
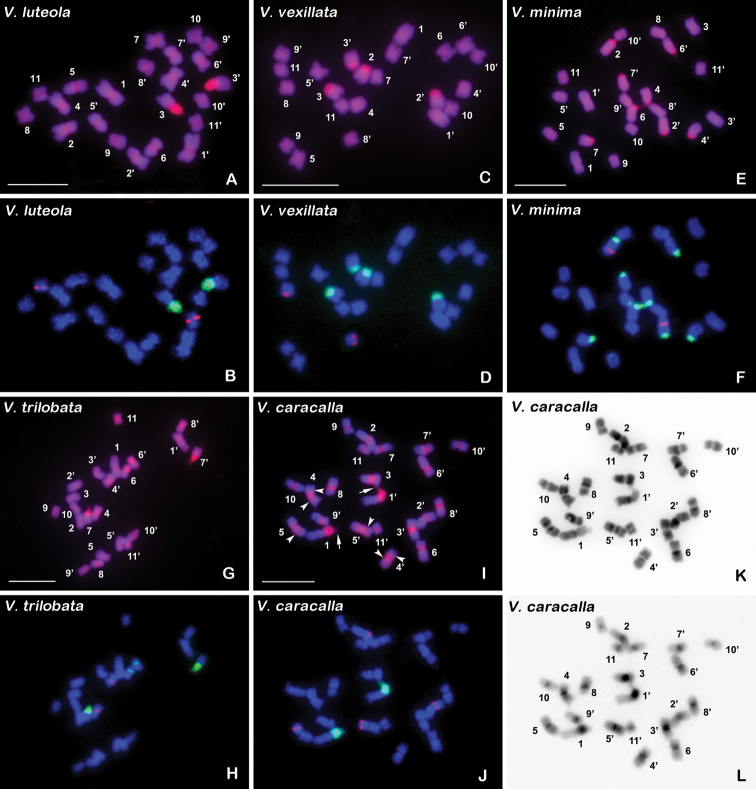
Mitotic chromosomes from *V. luteola* (**A, B**), *V. vexillata* (**C, D**), *V. minima* (**E, F**), *V. trilobata* (**G, H**), and *V. caracalla* (**I–L**) stained using CPD method and sequential dual-colour FISH with digoxigenin-labelled 5S and biotin-labelled 45S rDNA probes. **A, C, E, G, I** are the chromosomes stained using CPD. The chromosome numbers are designated by karyotyping. **B, D, F, H, J** are the chromosomes displaying the 5S (red) and 45S rDNA (green) signals. The total DNA was counterstained using DAPI (blue). **K, L** are DAPI and PI grey scale images of the *V. caracalla* chromosomes stained using CPD, respectively. The images are converted to reverse images with Photoshop software. Arrows and arrowheads in **I** indicate the satellites and interstitial CPD bands, respectively. Scale bars: 10 µm.

**Figure 2.**
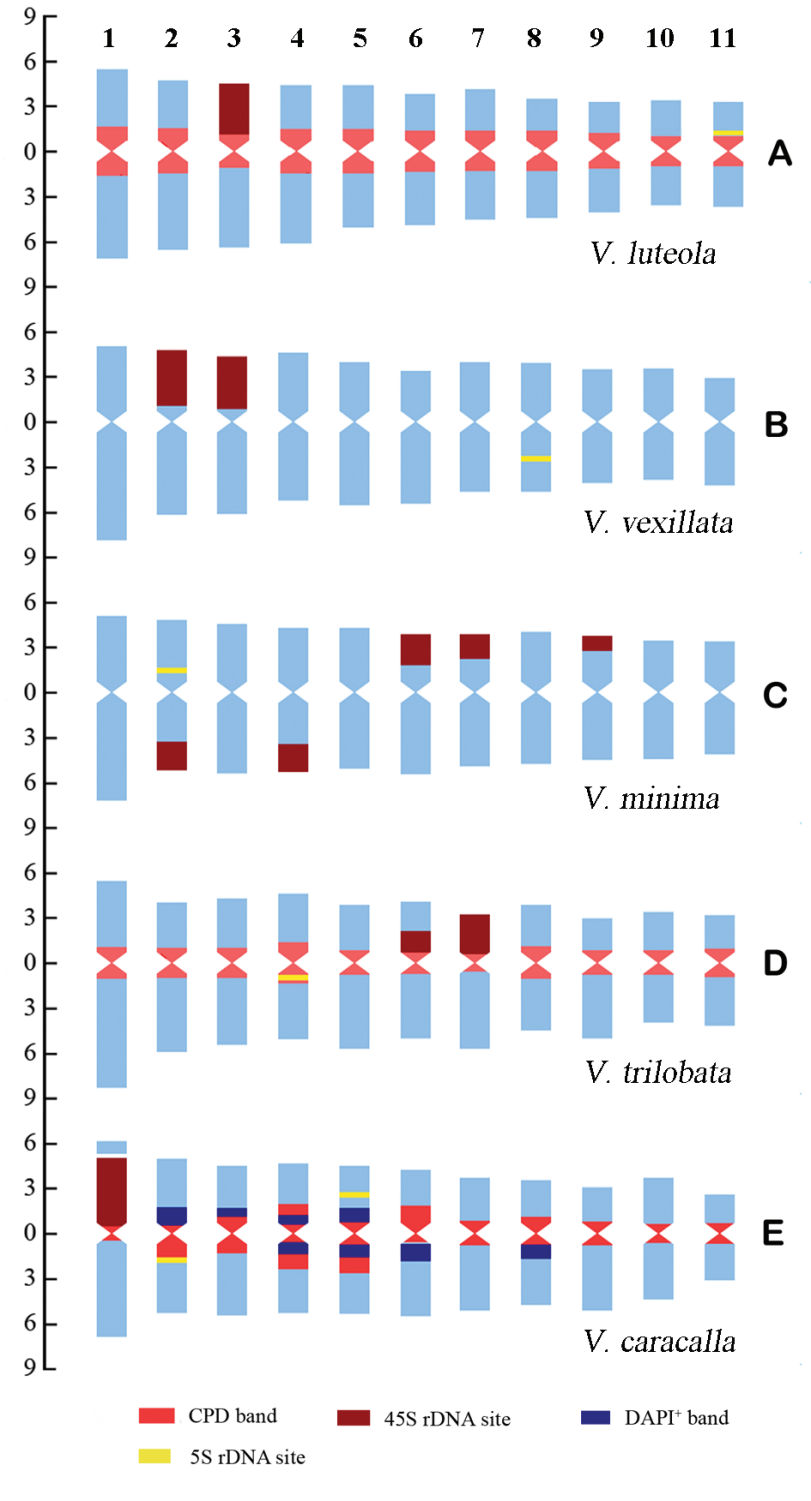
Idiograms of the five *Vigna* species that display the chromosome measurements, and the position and size of the fluorochrome bands and rDNA-FISH signals. **A–E** indicate *V. luteola*, *V. vexillata*, *V. minima*, *V. trilobata*, and *V. caracalla*, respectively. The ordinate scale on the left indicates the relative length of the chromosomes (i.e. % of haploid complement). The numbers at the top indicate the chromosomes 1 to 11.

Representative mitotic chromosomes of the five species studied are shown in Figure 1. The karyotypic parameters are listed in Table 1. The chromosome measurements for the five species are given in Suppl. material 1: Table S1. Idiograms displaying the chromosome measurements, position and size of the CPD bands and rDNA-FISH signals are illustrated in Figure 2.

**Table 1.** Karyotypic parameters of the five wild *Vigna* species (all, 2n = 2x = 22).

**Species**	**KF**	**TCL ± SE (μm)**	**C (μm)**	**RRL**	**CI±SE**	**A1**	**A2**	**As K (%)**	**AI**	**Stebinns’ types**
*V. luteola*	11m	33.81 ± 1.56	3.07	6.88–12.40	44.35 ± 2.45	0.20	0.21	55.97	1.15	1A
*V. vexillata*	11m	25.67 ± 2.02	2.33	6.99–12.66	43.24 ± 3.45	0.23	0.19	57.01	1.52	1A
*V. minima*	11m	38.29 ± 1.04	3.48	7.37–12.14	44.55 ± 2.03	0.19	0.14	55.53	0.66	1A
*V. trilobata*	9m + 2sm	36.56 ± 2.73	3.32	7.20–13.48	42.15 ± 3.87	0.27	0.19	58.00	1.76	1A
*V. caracalla*	10m (1SAT) + 1sm	46.62 ± 1.71	4.24	5.61–12.80	44.37 ± 3.13	0.20	0.20	55.39	1.41	1B

Notes: KF, Karyotype formula of haploid; TCL, total length of the haploid complement (i.e. karyotype length); C, mean chromosome length; SAT, satellite chromosome; RRL, ranges of chromosome relative length; CI, mean centromeric index; A1 and A2, the intrachromosomal asymmetry index and the interchromosomal asymmetry index of Romero Zarco (1986), respectively; AsK%, the ratio of length of all long arms in chromosome set to total chromosome length in set of Arano (1963); AI, the karyotype asymmetry index of Paszko (2006); Stebinns’ types, the karyotype asymmetry category of Stebbins (1971).

All the five *Vigna* species studied have diploid chromosome number 2n = 2x = 22. The metaphase chromosomes were small, with a mean chromosome length between 2.33 μm (*V. vexillata*) and 4.24 μm (*V. caracalla*). The total length of the haploid complement (TCL) ranged from 25.67 μm to 46.62 μm, and the mean centromeric index (CI) of the complements varied between 42.15 ± 3.87 (*V. trilobata*) and 44.55 ± 2.03 (*V. minima*). *V. caracalla* exhibited the most variation in chromosome length, and *V. trilobata* was characterized by the highest level of variation in the centromeric index.

The karyotypes of *V. luteola*, *V. vexillata*, *V. minima* were composed of metacentric (m) chromosomes only, while those of *V. trilobata* and *V. caracalla* were composed of metacentric and submetacentric (sm) chromosomes (Table 1, Suppl. material 1: Table S1; Fig. 2). In *V. caracalla*, the first chromosome pair had a satellite with secondary constriction (SC) that located at the distal position of the short arm (Figs 1I, 2E). All the karyotypes were quite symmetrical, falling into the Stebbins’ categories 1A or 1B (Table 1). The ranges of intrachromosomal asymmetry index (A1) and the interchromosomal asymmetry index (A2) were as follows: A1 = 0.19–0.27, and A2 = 0.14–0.21. The As K% ranged from 55.53 to 58.00, and the asymmetry index (AI) ranged from 0.66 to 1.76. According to the AI values, the karyotype of *V. minima* was the most symmetrical and that of *V. trilobata* was the most asymmetrical among the five taxa.

### Fluorochrome banding patterns

**Figure 3.**
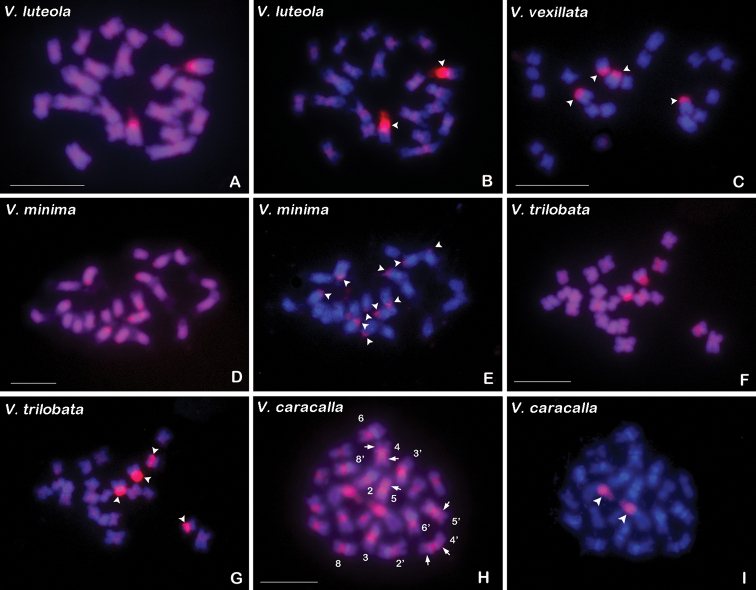
GISH with *Vigna umbellata* genomic DNA probe (red) to the chromosomes of *V. luteola* (**A, B**), *V. vexillata* (**C**), *V. minima* (**D, E**), *V. trilobata* (**F, G**), and *V. caracalla* (**H, I**). **A, D, F, H**CPD banded chromosomes before the hybridization procedure. The chromosomes shown in C is the same spread shown in Figure 1C, D. The chromosomes showing interstitial CPD bands and pericentromeric DAPI^+^ bands in H are numbered according to the karyotype of this species. Arrowheads in **B, C, E, G, I** indicate the signals corresponding to the 45S rDNA sites. Arrows in **H** indicate the interstitial CPD bands. Scale bars: 10 μm.

CPD staining revealed distinct heterochromatin differentiation among the five species studied (Figs 1–3; Table 2). Red CPD bands were shown in all species, but blue-fluorescent DAPI^+^ bands were shown only in *V. caracalla* (Figs 1I, 3H). The CPD bands were shown to be reverse PI-DAPI bands resulting from the intensity of the contrast between the PI (red) and DAPI (blue) fluorescence (Fig. 1I, K, L). In each species, all the chromosomal regions corresponding to the 45S rDNA sites, which were demonstrated by sequential FISH with rDNA probes, displayed CPD bands (Fig. 1A, C, E, G, I). All (peri) centromeric regions in *V. luteola*, *V. trilobata* and *V. caracalla* showed CPD bands (Figs 1A, G, I, 3A, F, H), while those in *V. vexillata* and *V. minima* did not show CPD bands (Figs 1C, E, 3D). In particular, the 5S rDNA sites in *V. minima* (Fig. 1E, F), and three pairs of interstitial sites (located in both short and long arms of chromosome pair 4, and the long arms of chromosome pair 5, respectively) in *V. caracalla* displayed CPD bands (Figs 1I, 3H). *V. caracalla* showed eight pairs of DAPI^+^ bands that occurred in the pericentromeric regions of the short arms of chromosome pairs 2, 3, 4 and 5, and the pericentromeric regions of the long arms of chromosome pairs 4, 5, 6 and 8 (Figs 1I, K, 3H). These DAPI^+^ bands were also shown in the DAPI-counterstained chromosomes after the FISH procedure (Figs 1J, 3I). The total amount of non-rDNACPD bands in *V. luteola*, *V. trilobata* and *V. caracalla* were 29.19%, 20.04%, and 21.68% of the karyotype length, respectively (Tables 2, Suppl. material 1: Table S1). The size of non-rDNACPD bands varied between the chromosome pairs in each species (Fig. 2; Suppl. material 1: Table S1). The total amount of DAPI^+^ bands in relation to the karyotype length was 8.19% in *V. caracalla* (Fig. 2; Suppl. material 1: Table S1).

### FISH patterns of 5S and 45S rDNA sites

FISH results of the 5S and 45S rDNA probes to the CPD-stained mitotic chromosomes are presented in Figure 1. The number and position of the rDNA sites are summarized in Table 2 and illustrated in Figure 2.

The FISH patterns of 5S and 45S rDNAs of the five species displayed conspicuous interspecific variation. Among the five taxa, *V. luteola*, *V. vexillata*, *V. minima* and *V. trilobata* had a single pair of 5S rDNA sites, while *V. caracalla* had two pairs of 5S sites (Figs 1B, D, F, H, J, 2A–E; Table 2). The 5S loci in *V. luteola* and *V. trilobata* were located in the pericentromeric regions of the relevant chromosome short or long arms, while those in *V. vexillata*, *V. minima* and *V. caracalla* were distributed in the interstitial regions of the short arms or long arms of the respective chromosomes. The 5S locus in *V. trilobata* was colocalized with a portion of the pericentromeric CPD bands (Figs 1G, H, 2D). With regard to length, the chromosome pair bearing the 5S locus in *V. luteola* was the shortest in the complement, and the 5S-bearing chromosome pairs in other four species were of an intermediate size.

For the 45S rDNA sites, there was considerable variation in number, size and position among the five taxa analyzed (Table 2). The number of 45S rDNA loci varied as follows: one in *V. luteola* and *V. caracalla*, two in *V. vexillata* and *V. trilobata* and five in *V. minima* (Figs 1B, D, F, H, J, 2A–E). In *V. luteola*, the single 45S locus comprised the entire short arms of chromosome pair 3 (Figs 1A, B, 2A). The two 45S loci in *V. vexillata* were terminally located on the short arms of pairs 2 and 3 and occupied the majority of the arms (Figs 1C, D, 2B). All the five 45S loci in *V. minima*, four major and one minor loci, were terminally located on the long or short arms of chromosome pairs 2, 4, 6, 7 and 9, among which the locus on pair 2 was syntenic to the 5S locus (Figs 1E, F, 2C). In *V. trilobata*, one major locus occupied the entire short arms of pair 7, and one minor locus was pericentromerically placed on the short arms of pair 6 (Figs 1G, H, 2D). The single 45S locus in *V. caracalla* occupied the entire short arms of pair 1 except the satellites (Figs 1I, J, 2E). The 45S rDNA chromatin of the terminal loci in the five taxa accounted for 32.06–100% of the arm length (calculated from the size of the rDNACPD bands; Table 2).

**Table 2.** The distribution of fluorochrome bands and rDNA sites in the five wild *Vigna* species.

**Species**	**Fluorochrome bands**				**Number (pairs) and location of rDNA sites^†^**	
	**Type**	**Distribution^†^**	**Amount (%)^‡^**	**Band size (mean)^§^**	**5S^|^**	**45S^|^**
*V. luteola*	CPD	all CENs, PCENs and 45S sites	29.19	1.98–3.21 (2.65)	one [11S-PCEN (16.55%)]	one (3S)
*V. vexillata*	CPD	all 45S sites			one [(8L-INT(52.29%)]	two [2S-TER (20.53%), 3S-TER (16.73%)]
*V. minima*	CPD	all 45S and 5S sites			one [2S-INT (30.86%)]	five [2L-TER (58.64%), 4L-TER (59.42%), 6S-TER (38.91%), 7S-TER (50.74%), 9S-TER (67.94%)]
*V. trilobata*	CPD	all CENs, PCENs and 45S sites	20.04	2.73–1.12 (1.82)	one [4L-PCEN(14.95%)]	two [6S-PCEN(25.05%), 7S]
*V. caracalla*	CPD	all CENs, PCENs and 45S sites, 4S-, 4L-, 5L-INTs	21.68^¶^	0.89–2.63 (1.55)	two [2L-INT(34.32%), 5S-INT (56.7%)]	one (1S)
	DAPI	2, 3, 4, 5S-PCENs; 4, 5, 6, 8L-PCENs	8.19	0.69–1.38 (1.04)		

^†^S and L represent short and long arms, respectively; CEN, PCEN, INT and TER represent centromeric, pericentromeric, interstitial, terminal position, respectively; figures ahead of the positions are the designations of the chromosome pair involved. ^‡^Amount of bands in the genome expressed as percentage of the karyotype length (rDNACPD bands are excluded). ^§^The percentage of the size of the bands of each chromosome pair in relation to the karyotype length. ^|^The percentages in square brackets are the percentage distance from centromere to the rDNA site (*di* = *d*× 100/*a*; *d* = distance of starting point of terminal sites judged by CPD bands or center of non-terminal sites judged by the FISH signals from the centromere, *a* = length of the corresponding chromosome arm). ^¶^The value consists of the amounts of centromeric and pericentromeric CPD bands (18.29%) as well as interstitial CPD bands (3.39%).

### cGISH signal patterns

Comparative genomic *in situ* hybridization with *V. umbellata* genomic DNA probe was employed to reveal the homology of repetitive DNA sequences between *V. umbellata* and the five wild *Vigna* species (Fig. 3). The genomic probe produced 45S rDNA signals in all species and non-rDNA signals in *V. luteola* and *V. trilobata* (Fig. 3B, G). All 45S sites were strongly labeled by the genomic DNA probe in the five species (Fig. 3B, C, E, G, I). In *V. luteola*, expect for the 45S signals, weak signals were generated in the proximal regions of the two arms of each chromosome (Fig. 3A, B), while in *V. trilobata*, weak non-rDNA signals were mainly concentrated in all (peri)centromeric regions, which basically corresponded to the (peri)centromeric CPD bands (Fig. 3F, G).

## Discussion

### Karyotype variation

In the current study, detailed karyotypes of *V. luteola*, *V. vexillata*, *V. minima*, *V. trilobata* and *V. caracalla* are established using a dataset of chromosome measurements, fluorochrome bands, and rDNA-FISH signals, thus providing the first primary molecular cytogenetic characterization of these wild *Vigna* species. Although FISH mapping of rDNAs in V. vexillata var. tsusimensis Matsumura, 1902 has been conducted (Chio et al. 2013), but the detailed karyotype of this species has not yet been established. Our results reveal that the karyotypic parameters and patterns of the fluorochrome bands and rDNA sites vary among the five *Vigna* species studied, enabling an accurate distinguishment between individual genomes.

This study identifies the chromosome number of all the five species as 2n = 22, in accordance with that reported previously by other authors (Sen and Bhowal 1960; Joseph and Bouwkamp 1978; Rao and Chandel 1991; Galasso et al. 1993; Venora and Saccardo 1993; Venora et al. 1999; Shamurailatpam et al. 2012, 2016; Choi et al. 2013). The conventional karyotypes of the five species studied here have been reported by earlier workers (Joseph and Bouwkamp 1978; Rao and Chandel 1991; Venora et al. 1999; Shamurailatpam et al. 2016). However, the published karyotype formulae of *V. minima* (Shamurailatpam et al. 2016), *V. trilobata* (Rao and Chandel 1991) and *V. caracalla* (Joseph and Bouwkamp 1978) were not comparable because the chromosomes were not classified according to the system of Levan et al. (1964). The current karyotypes of *V. luteola* and *V. vexillata*, n = 11m, are more symmetric than the karyotypes reported by Venora et al. (1999), which were comprised of both metacentric and submetacentric chromosomes. This discrepancy is probably due to difference in the accessions analysed, and difficulty in identifying chromosomes using the classical staining technique in the previous studies.

The results reveal significant variation in karyotype length (TCL) among the five taxa studied. For example, the TCL of *V. caracalla* was 1.82 times longer than that of *V. vexillata*. Except *V. caracalla*, the TCLs of the other four wild species were much shorter than those of the seven cultivated *Vigna* species obtained previously by us (She et al. 2015). With respect to the karyotype asymmetry (according to the AI values), among the five wild and seven cultivated *Vigna* species that has been studied using molecular cytogenetic method, *V. minima* and *V. subterranea* have the lowest asymmetry; *V. radiata*, V. mungo var. mungo and *V. aconitifolia* have the most asymmetric; *V. luteola*, *V. vexillata*, *V. trilobata*, *V. caracalla*, V. unguiculata ssp. sesquipedalis, *V. angularis* and *V. umbellata* are intermediately asymmetric (She et al. 2015).

### Heterochromatin differentiation

The significant variation in CPD and DAPI^+^ bands, with regard to appearance, position and size, reflects distinct GC-rich and AT-rich heterochromatin differentiation among the five wild *Vigna* species (She et al. 2006; She and Jiang 2015). Similar heterochromatin differentiation has been observed among the seven cultivated *Vigna* species (She et al. 2015). As we know, heterochromatic blocks are chromosomal regions that contain a high density of satellite DNA and transposable elements (Heslop-Harrison and Schwarzacher 2011). These facts indicate that alterations in repeated DNA sequences have contributed to the karyotypic differentiation during the diversification of *Vigna* species (de Moraes et al. 2007; Hamon et al. 2009; Robledo et al. 2009; Mondin and Aguiar-Perecin 2011; She et al. 2015; Amosova et al. 2017).

With the exception of the rDNACPD bands, *V. luteola*, *V. trilobata*, and *V. caracalla* also displayed centromeric and pericentromeric non-rDNACPD bands. Especially, *V. caracalla* possessed interstitial non-rDNACPD bands, which have not been observed in other *Vigna* species (She et al. 2015). Centromeric, pericentromeric or proximal GC-rich heterochromatin without colocalization with rDNA sites have been observed by using CPD or CMA/DAPI staining on the chromosomes of the seven cultivated *Vigna* species (de A Bortoleti et al. 2012; She et al. 2015) as well as many other Phaseoloid species such as the two cultivated *Canavalia* (Adanson, 1763) species (She et al. 2017), *Crotalaria* (Linnaeus, 1753) species of *Calycinae* and *Crotalaria* sections (Mondin and Aguiar-Perecin 2011), *Lablab purpureus* (Linnaeus, 1753) Sweet, 1826 (She and Jiang 2015), the four cultivated *Phaseolus* (Linnaeus, 1753) species (Bonifácio et al. 2012) and *Psophocarpus tetragonolobus* (Linnaeus, 1753) Candolle, 1825 (Chaowen et al. 2004). These facts suggest that the existence of (peri)centromeric GC-rich heterochromatin is an ancestral genome feature that occurred before the divergence of the Phaseoloid clade of the subfamily Papilionoideae (LPWG 2013). However, the inexistence of non-rDNA GC-rich heterochromatin in *V. vexillata* and *V. minima* seems to be in contradiction with this speculation. A reasonable explanation is that the non-rDNA GC-rich heterochromatin of these two species has undergone a reduction of GC content after speciation, resulting in the disappearance of red CPD bands (She et al. 2006). The changes of non-rDNACPD bands in amount, distribution, and GC content have been observed among the seven cultivated *Vigna* species. For example, in *V. radiata*, non-rDNA GC-rich heterochromatin blocks disappeared from five pairs of chromosomes; in *V. mungo*, non-rDNA GC-rich heterochromatin blocks occurred only in the proximal regions of the long arms of eight pairs of chromosomes (She et al. 2015). As for the GC-rich regions corresponded to the 5S rDNA sites that observed in *V. minima*, the variation in the base composition of the non-transcribed spacer (NTS) of the 5S rDNA repeats or the interspersion of other GC-rich repeated DNAs with the 5S rDNA repeats may explain it (Cabral et al. 2006; Hamon et al. 2009).

The occurrence of the pericentromeric DAPI^+^ bands in *V. caracalla* was another conspicuous heterochromatic differentiation of this species. Among the *Vigna* species previously analyzed by fluorochrome banding technique, AT-rich heterochromatin blocks have been observed in the pericentromeric regions of several chromosome pairs of *V. radiata* (de A Bortoleti et al. 2012; She et al. 2015). The AT-rich heterochromatin in *V. radiata* and *V. caracalla* should arise after the divergence of *Vigna* species because of its non-universality.

### Variation of rDNA loci

To date, FISH mapping of rDNA sites has been reported only for V. vexillata var. tsusimensis among the wild species within the genus *Vigna* (Choi et al. 2013). Regarding the number and position of rDNA loci of this species, our findings is significantly different from the previous report, in which three pairs of 45S loci and two pairs of 5S loci were observed (Choi et al. 2013). The identified divergence could be due to the difference in the accessions analysed.

Our rDNA-FISH results reveal considerable variations in number, position and even size of both 45S and 5S rDNA sites among the five wild *Vigna* species studied. Similarly, wide interspecific differences in the pattern of rDNA sites were observed among the seven cultivated *Vigna* species (She et al. 2015). Inferring from the rDNA-FISH data of the twelve *Vigna* species investigated by us, the FISH patterns of the 45S rDNA sites in species of this genus were more polymorphic than those of the 5S rDNA. This phenomenon has been reported in many different plant genera such as *Phaseolus* Linnaeus, 1753 (Moscone et al. 1999), *Paeonia* Linnaeus, 1753 (Zhang and Sang 1999), *Brassica* Linnaeus, 1753 (Hasterok et al. 2001), *Oryza* Linnaeus, 1753 (Chung et al. 2008), *Coffea* Linnaeus, 1753 (Hamon et al. 2009), *Brachypodium* P. Beauvois, 1812 (Wolny and Hasterok 2009), *Citrullus* Schrader ex Ecklon & Zeyher, 1836 (Li et al. 2016) and *Allium* Linnaeus, 1753 (Maragheh et al. 2019). The interspecies and intraspecific variations in the number and location of rDNA sites has been attributed to various mechanisms such as transposon-mediated transposition, homologous and/or non-homologous unequal crossing over, inversion, translocation and locus duplication/deletion (Moscone et al. 1999; Zhang and Sang 1999; Datson and Murray 2006; Pedrosa-Harand et al. 2006; Chung et al. 2008; Raskina et al. 2008; Weiss-Schneeweiss et al. 2008). The differentiation in the chromosomal organization of rDNA clusters between plant species was generally correlated with the chromosome evolution during speciation (Datson and Murray 2006; Moscone et al. 2007; Raskina et al. 2008; Weiss-Schneeweiss et al. 2008). Among the five taxa studied the number of 5S loci is rather conserved: four species had a single 5S locus located in pericentromeric or interstitial regions. Similarly, five of the seven cultivated *Vigna* species had only one 5S locus that was located in the proximal, interstitial, pericentromeric or centromeric regions (She et al. 2015). Furthermore, among the twelve species that were investigated using molecular cytogenetic approaches by us, the single 5S locus in *V. luteola*, *V. umbellata* and *V. aconitifolia* and one 5S locus in *V. radiata* were located in the pericentromeric, centromeric, or proximal regions of the short arms of the shortest chromosome pair (She et al. 2015). These facts suggest that the ancestral progenitor of the genus *Vigna* bear a single 5S locus that is located on the short arms of the shortest chromosomes in the complement. Chromosome rearrangements such as inversion and translocation may change the position of the 5S locus or produce longer 5S-bearing chromosomes (Moscone et al. 2007; Chung et al. 2008; Weiss-Schneeweiss et al. 2008; She et al. 2015). The increased number of 5S loci in *V. caracalla* probably originated from the transposition of the 5S rDNA (Raskina et al. 2008). As for 45S site, one, two, three, four and five loci were identified in the twelve *Vigna* species studied by us, respectively (She et al. 2015). A total of thirty-one 45S loci were detected in the twelve species, among which twenty-four were terminal and seven were pericentromeric. Considering that *V. aconitifolia* and *V. luteola* had a single terminal 45S locus and the Aconitifoliae section was the ancestral section within the subgenus Ceratotropis (Doi et al. 2002), the ancestral progenitor genome of *Vigna* species might bear a single terminal 45S locus. Another terminal 45S locus in *V. vexillata*, and the other four terminal 45S loci in *V. minima* might result from one or more non-homologous unequal crossing over between the terminal chromosomal regions (Zhang and Sang 1999; Pedrosa-Harand et al. 2006). The pericentromeric 45S locus in *V. trilobata*, like the pericentromeric locus in V. unguiculata subsp. sesquipedalis (Linnaeus, 1753) Verdcourt 1970, and three pericentromeric 45S loci in *V. umbellata* (She et al. 2015), might originate from transposition of the terminal 45S rDNA cluster (Datson and Murray 2006; Chung et al. 2008; Raskina et al. 2008).

### Phylogenetic relationships

In the early time, the *Vigna* genus was divided into seven subgenera (Maréchal et al. 1981). Delgado-Salinas et al. (2011) proposed, based on phylogenetic analysis of cpDNA *trnK* and nuclear ribosomal ITS/5.8S (ITS) sequence variation, a new circumscription of *Vigna* Savi sensu stricto, which includes five subgenera, *Ceratotropis*, *Haydonia*, *Lasiospron*, *Plectrotropis*, and *Vigna*, of the seven recognized by Maréchal et al. (1981). The Vigna subg. Sigmoidotropis of Maréchal et al. (1981), in which *V. caracalla* was previously placed, is now divided into six genera, *Ancistrotropis* A. Delgado, 2011, *Cochliasanthus* Trew, 1764, *Condylostylis* Piper, 1926, *Leptospron* (Benth. and Hook.f., 1865) A. Delgado, 2011, *Helicotropis* A. Delgado, 2011, and *Sigmoidotropis* (Piper, 1926) A. Delgado, 2011 (Delgado-Salinas et al. 2011). *V. caracalla* is transferred to the monotypic genus *Cochliasanthus*, and named as *Cochliasanthus caracalla*. Our molecular cytogenetic karyotyping data revealed that this species had several distinct characteristics compared to the other eleven *Vigna* species studied by us: existence of several interstitial CPD bands, pericentromeric DAPI bands, as well as satellites associated with the short arms that consist of 45S rDNA clusters (She et al. 2015). These facts indicate that *V. caracalla* significantly differentiates from other *Vigna* species at chromosome level, supporting the taxonomic separation of *V. caracalla* from the genus *Vigna* (Delgado-Salinas et al. 2011).

Among the remaining four wild *Vigna* species analyzed, both *V. luteola* and *V. vexillata* are of African origin being categorized into Vigna subg. Vigna and subg. Haydonia, respectively (Delgado-Salinas et al. 2011), while both *V. minima* and *V. trilobata* are Asiatic Vigna (subg. Ceratotropis) species, and belong to Section Angulares and Section Aconitifoliae, respectively (Doi et al. 2002; Goel et al. 2002; Javadi et al. 2011). The molecular phylogeny of *Vigna* has been investigated intensively using sequence data from the rDNAITS, the IGS of 5S rDNA, and chloroplast DNA (Doi et al. 2002; Goel et al. 2002; Tun and Yamaguchi 2007; Saini et al. 2008; Saini and Jawali 2009; Delgado-Salinas et al. 2011; Javadi et al. 2011; She et al. 2015; Raveenadar et al. 2018). Here the molecular phylogenies revealed by other authors and the molecular cytogenetic data obtained by us are combined to analyze the phylogenetic relationships among the wild and cultivated *Vigna* species studied molecular-cytogenetically by us. The molecular phylogenetic trees inferred from cpDNA *trnK* and nrDNAITS sequence by Delgado-Salinas et al. (2011) revealed that *V. luteola* and *V. subterranea* were included within the same group of one African *Vigna* subclade and belonged to different subgroups, while *V. vexillata* and *V. unguiculata* were included within the same group of another African *Vigna* subclade and placed at different subgroups; *V. minima*, *V. umbellata* and *V. angularis* were included within one subclade of the subg. Ceratotropis clade and clustered into three different subgroups, while *V. trilobata* and *V. aconitifolia* were included within another subclade of subg. Ceratotropis clade and clustered into different subgroups. Similar phylogenetic relationships among these species mentioned above were also revealed using the IGS of 5S rDNA (Saini and Jawali 2009), and the sequences of *rbcL* + *psbA-trnH* + ITS2 + *matK* region (Raveenadar et al. 2018). Our previous rDNA-FISH revealed that V. *subterranea* had two terminal and one pericentromeric 45S loci, and a single interstitial 5S locus located on a medium-sized chromosome pair (She et al. 2015), being significantly different from the rDNA distribution pattern of *V. luteola*. Especially, non-rDNAcGISH signals of *V. umbellata* genomic DNA probe were produced in *V. luteola* but not in *V. subterranea*. These facts suggest that there is significant genome differentiation between *V. luteola* and *V. subterranea*, in disagreement with the molecular phylogeny. Specially must point out in here, the production of non-rDNAcGISH signals on the chromosomes of *V. luteola* with *V. umbellata* genomic DNA probe was perplexing because *V. luteola* and *V. umbellata* belong to different subgenera and should be relatively distantly related (Delgado-Salinas et al. 2011). To solve this puzzling problem, more *V. luteola* accessions need to be studied using FISH. Our molecular cytogenetic data also revealed prominent differentiation between *V. vexillata* and *V. unguiculata* because, compared to *V. unguiculata*, *V. vexillata* lacked (peri)centromeric GC-rich regions and had less number of 45S and 5S loci (de A Bortoleti et al. 2012; She et al. 2015). The reported molecular phylogenies showed that *V. minima* and *V. umbellata*, *V. trilobata* and *V. aconitifolia* were closely related, respectively (Doi et al. 2002; Goel et al. 2002; Saini and Jawali 2009; Delgado-Salinas et al. 2011). Our molecular cytogenetic data support the close relationship between *V. trilobata* and *V. aconitifolia* because both of them had (peri)centromeric CPD bands, similar 45S-bearing chromosome pair (pair 7 and pair 4 in *V. trilobata* and *V. aconitifolia*, respectively), and pericentromeric cGISH signals of *V. umbellata* genomic DNA probe (She et al. 2015). However, the close relationship between *V. minima* and *V. umbellata* was not confirmed by the molecular cytogenetic data because *V. minima* lacked cGISH signals of *V. umbellata* genomic DNA probe, and (peri)centromeric CPD bands which existed in all (peri)centromeric regions of *V. umbellata* (She et al. 2015). In summary, our molecular cytogenetic data not only partially support the molecular phylogenetic relationships between related *Vigna* species, but also reveal considerable genome differentiation between the *Vigna* species that have been proved to be closely related by molecular phylogenetic analysis. It is necessary to clarify the conflicts between the molecular phylogenies and molecular cytogenetic data by performing integrated study of molecular phylogenetic and molecular cytogenetic analyses using more accessions of related *Vigna* species.

## Conclusions

Molecular cytogenetic karyotypes of five wild *Vigna* species, *V. luteola*, *V. vexillata*, *V. minima*, *V. trilobata* and *V. caracalla* are established for the first time using fluorochrome banding and rDNA-FISH techniques. Comparative molecular cytogenetic karyotyping reveals distinct variations in the karyotypic parameters, and the patterns of the fluorochrome bands and rDNA sites among species, enabling an accurate distinguishment between individual genomes. The molecular cytogenetic data of the five species is helpful to clarify the phylogenetic relationships among related *Vigna* species.

## Supplementary materials

Supplementary material 1 Table S1. Chromosome measurements of the five wild *Vigna* species obtained from five metaphases per species Data type species data https://binary.pensoft.net/file/425682This dataset is made available under the Open Database License (http://opendatacommons.org/licenses/odbl/1.0/). The Open Database License (ODbL) is a license agreement intended to allow users to freely share, modify, and use this Dataset while maintaining this same freedom for others, provided that the original source and author(s) are credited.Chao-Wen She, Ying Mao, Xiang-Hui Jiang, Chun-Ping He
