# Supplementary material for: Comparative molecular cytogenetic characterization of five wild Vigna species (Fabaceae)
Source: Comp Cytogenet. 2020 Jun 26;14(2):243–64. doi: 10.3897/CompCytogen.v14i2.51154 (PMC7334243; doi:10.3897/CompCytogen.v14i2.51154)
Supplement: Supplementary material 1 — Table S1. Chromosome measurements of the five wild Vigna species obtained from five metaphases per species [file comparative_cytogenetics-14-243-s001.doc]

**Table S1. Chromosome measurements of the five wild *Vigna* species obtained from five metaphases per species.**

| Species | Chr. No. | Relative length (%) | | | Arm ratio ± SD | Type | (Peri)centromeric CPD band size ± SD^‡^ |
| --- | --- | --- | --- | --- | --- | --- | --- |
|  |  | Short arm ± SD | Long arm ± SD | Total ± SD |  |  |  |
| *V. luteola* | 1 | 5.35 ±0.22 | 7.05±0.16 | 12.40±0.29 | 1.32±0.06 | m | 3.21±0.27 |
|  | 2 | 4.66 ±0.34 | 6.49±0.26 | 11.15±0.59 | 1.40±0.06 | m | 3.03±0.30 |
|  | 3 | 4.40 ±0.10 | 6.32±0.16 | 10.72±0.20 | 1.44±0.04 | m | 2.13±0.27 |
|  | 4 | 4.32±0.42 | 6.03±0.19 | 10.35±0.53 | 1.41±0.12 | m | 2.99±0.36 |
|  | 5 | 4.34±0.25 | 5.04±0.28 | 9.38±0.41 | 1.16±0.08 | m | 3.05±0.36 |
|  | 6 | 3.72±0.16 | 4.87±0.46 | 8.59±0.41 | 1.31±0.16 | m | 2.79±0.30 |
|  | 7 | 4.02±0.17 | 4.49±0.24 | 8.50±0.21 | 1.12±0.10 | m | 2.81±0.37 |
|  | 8 | 3.45±0.28 | 4.42±0.18 | 7.87±0.43 | 1.29±0.07 | m | 2.74±0.53 |
|  | 9 | 3.21±0.51 | 4.02±0.14 | 7.23±0.50 | 1.27±0.20 | m | 2.43±0.04 |
|  | 10 | 3.33±0.28 | 3.59±0.37 | 6.92±0.61 | 1.08±0.07 | m | 2.03±0.33 |
|  | 11 | 3.23±0.17 | 3.65±0.31 | 6.88±0.41 | 1.13±0.09 | m | 1.98±0.36 |
|  | Total | 44.03±1.14 | 55.97±1.14 | 100.00 |  |  | 29.19±2.85 |
| *V. vexillata* | 1 | 4.91±0.62 | 7.75±0.31 | 12.66±0.38 | 1.60±0.28 | m |  |
|  | 2 | 4.65±0.31 | 6.08±0.40 | 10.73±0.52 | 1.31±0.12 | m |  |
|  | 3 | 4.26±0.37 | 6.04±0.83 | 10.31±0.61 | 1.43±0.29 | m |  |
|  | 4 | 4.52±0.47 | 5.14±0.04 | 9.65±0.51 | 1.14±0.12 | m |  |
|  | *5* | 3.87±0.50 | 5.50±0.46 | 9.37±0.67 | 1.44±0.21 | m |  |
|  | *6* | 3.31±0.05 | 5.38±0.59 | 8.68±0.64 | 1.62±0.15 | m |  |
|  | *7* | 3.93±0.35 | 4.55±0.16 | 8.48±0.32 | 1.17±0.12 | m |  |
|  | *8* | 3.83±0.27 | 4.59±0.22 | 8.43±0.10 | 1.21±0.14 | m |  |
|  | *9* | 3.42±0.15 | 3.98±0.36 | 7.40±0.44 | 1.17±0.10 | m |  |
|  | 10 | 3.49±0.28 | 3.82±0.27 | 7.30±0.54 | 1.10±0.03 | m |  |
|  | 11 | 2.81±0.25 | 4.18±0.32 | 6.99±0.52 | 1.49±0.11 | m |  |
|  | Total | 42.99±1.19 | 57.01±1.19 | 100.00 |  |  |  |
| *V. minima* | 1 | 5.00±0.37 | 7.13±0.32 | 12.14±0.43 | 1.43±0.13 | m |  |
|  | 2 | 4.73±0.07 | 5.08±0.36 | 9.81±0.29 | 1.08±0.09 | m |  |
|  | 3 | 4.46±0.07 | 5.33±0.35 | 9.79±0.42 | 1.19±0.06 | m |  |
|  | 4 | 4.21±0.17 | 5.20±0.22 | 9.41±0.29 | 1.24±0.07 | m |  |
|  | 5 | 4.18±0.17 | 5.02±0.09 | 9.21±0.19 | 1.20±0.05 | m |  |
|  | 6 | 3.78±0.50 | 5.39±0.39 | 9.16±0.22 | 1.45±0.27 | m |  |
|  | 7 | 3.78±0.31 | 4.86±0.65 | 8.64±0.34 | 1.30±0.29 | m |  |
|  | 8 | 3.92±0.02 | 4.72±0.08 | 8.63±0.10 | 1.20±0.02 | m |  |
|  | 9 | 3.72±0.09 | 4.37±0.28 | 8.09±0.38 | 1.18±0.05 | m |  |
|  | 10 | 3.38±0.05 | 4.36±0.24 | 7.73±0.29 | 1.29±0.05 | m |  |
|  | 11 | 3.31±0.31 | 4.06±0.24 | 7.37±0.37 | 1.24±0.14 | m |  |
|  | Total | 44.47±0.63 | 55.53±0.63 | 100.00 |  |  |  |
| *V. trilobata* | 1 | 5.32±0.20 | 8.16±0.39 | 13.48±0.31 | 1.54±0.12 | m | 2.07±0.34 |
|  | 2 | 3.92±0.25 | 5.85±0.34 | 9.76±0.36 | 1.50±0.15 | m | 2.00±0.15 |
|  | 3 | 4.20±0.20 | 5.39±0.18 | 9.59±0.26 | 1.28±0.08 | m | 1.94±0.49 |
|  | 4 | 4.52±0.26 | 5.01±0.34 | 9.53±0.60 | 1.11±0.02 | m | 2.73±0.39 |
|  | 5 | 3.80±0.41 | 5.60±0.04 | 9.40±0.43 | 1.49±0.16 | m | 1.61±0.38 |
|  | 6 | 4.00±0.15 | 4.95±0.10 | 8.95±0.22 | 1.24±0.04 | m | 1.41±0.04 |
|  | 7 | 3.16±0.17 | 5.65±0.24 | 8.81±0.08 | 1.79±0.17 | sm | 1.12±0.27 |
|  | 8 | 3.79±0.10 | 4.41±0.38 | 8.19±0.45 | 1.16±0.08 | m | 2.15±0.37 |
|  | 9 | 2.89±0.17 | 4.95±0.25 | 7.84±0.09 | 1.72±0.19 | sm | 1.51 ±0.27 |
|  | 10 | 3.32±0.28 | 3.93±0.21 | 7.25±0.49 | 1.18±0.04 | m | 1.68±0.38 |
|  | 11 | 3.09±0.14 | 4.11±0.18 | 7.20±0.33 | 1.33±0.00 | m | 1.82±0.28 |
|  | Total | 42.00±0.69 | 58.00±0.69 | 100.00 |  |  | 20.04±2.98 |
| *V.* *caracalla* | 1 | 6.06±0.59 | 6.74±0.66 | 12.80±1.21 | 1.11±0.06 | m^†^ | 0.92±0.09 |
|  | 2 | 4.87±0.06 | 5.19±0.12 | 10.05±0.16 | 1.07±0.02 | m | 2.11±0.40 |
|  | 3 | 4.42±0.22 | 5.40±0.46 | 9.82±0.58 | 1.22±0.10 | m | 2.60±0.29 |
|  | 4 | 4.54±0.26 | 5.24±0.25 | 9.77±0.46 | 1.16±0.06 | m | 1.43±0.32 |
|  | 5 | 4.41±0.19 | 5.25±0.22 | 9.66±0.12 | 1.19±0.10 | m | 1.46±0.37 |
|  | 6 | 4.11±0.13 | 5.37±0.12 | 9.48±0.24 | 1.31±0.02 | m | 2.35±0.22 |
|  | 7 | 3.64±0.48 | 5.06±0.64 | 8.71±0.21 | 1.42±0.36 | m | 1.58±0.25 |
|  | 8 | 3.45±0.31 | 4.66±0.37 | 8.11±0.67 | 1.35±0.04 | m | 1.77±0.14 |
|  | 9 | 2.97±0.34 | 5.05±0.25 | 8.02±0.18 | 1.72±0.27 | sm | 1.51±0.30 |
|  | 10 | 3.62±0.36 | 4.34±0.16 | 7.96±0.46 | 1.20±0.11 | m | 1.22±0.16 |
|  | 11 | 2.52±0.07 | 3.09±0.11 | 5.61±0.17 | 1.23±0.01 | m | 1.33±0.35 |
|  | Total | 44.61±0.17 | 55.39±0.17 | 100.00 |  |  | 18.29±2.38 |

Notes: SD, standard deviation. m, metacentric; sm, submetacentric. ^†^ satellite chromosome, the length of the satellite was included in the chromosome length, but the length of the secondary constriction was excluded. ^‡^ the percentage of the size of the band in relation to the karyotype length.
